# Supplementary figures and images for: Profiling of Bacterial and Fungal Microbial Communities in Cystic Fibrosis Sputum Using RNA
Source: mSphere. 2018 Aug 8;3(4):e00292-18. doi: 10.1128/mSphere.00292-18 (PMC6083091; doi:10.1128/mSphere.00292-18)

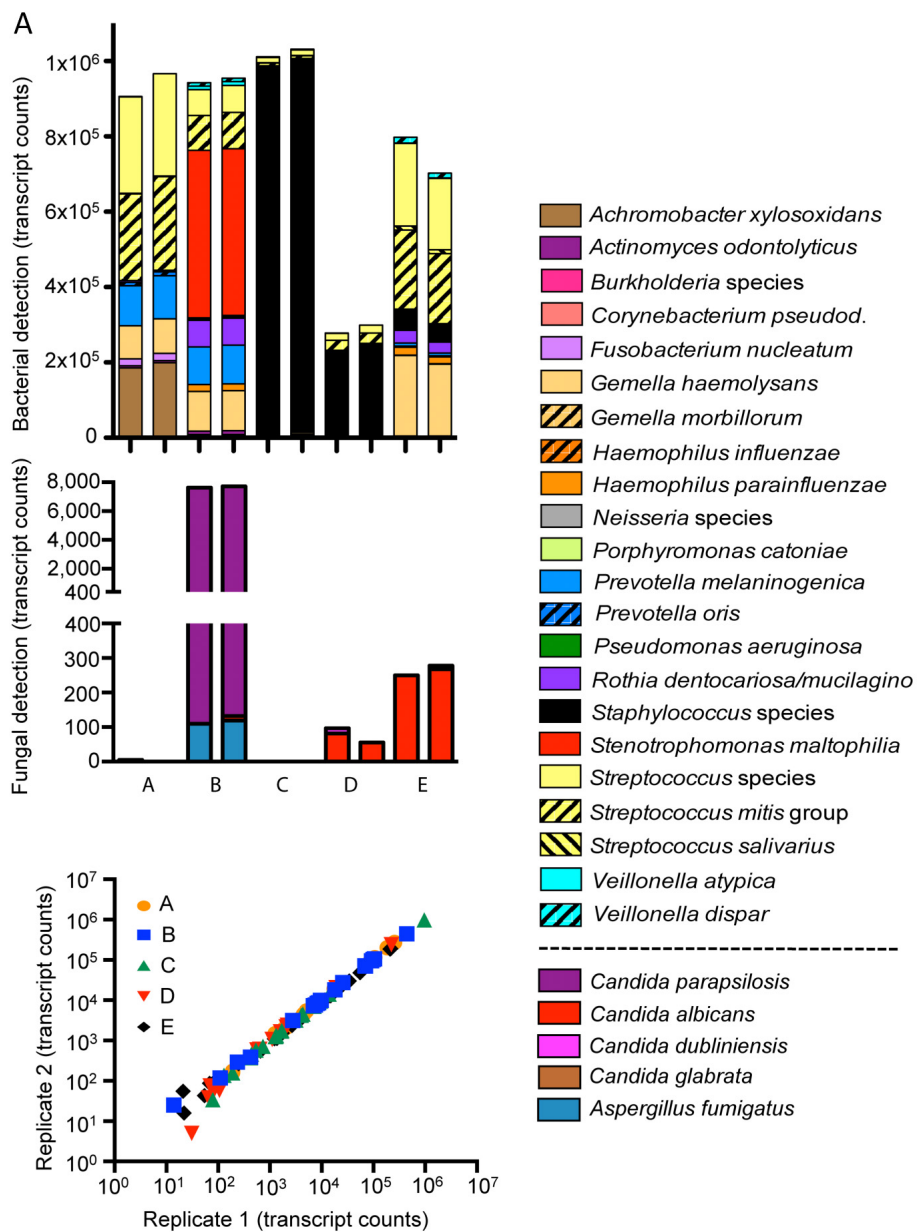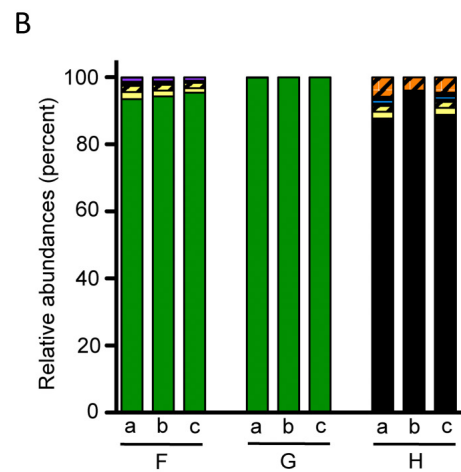

Supplement: FIG S1 [file sph004182610sf1.pdf]

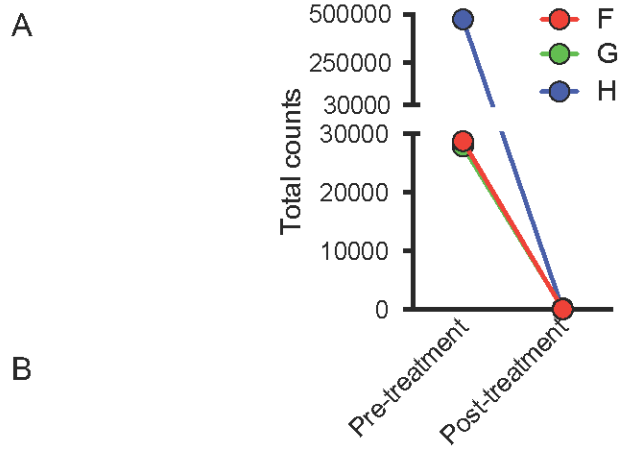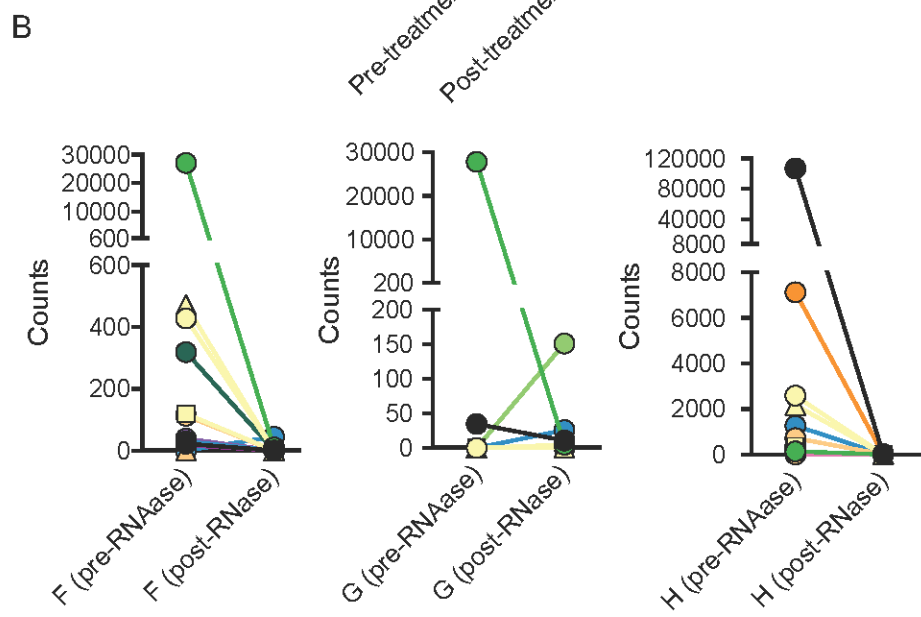

Supplement: FIG S2 [file sph004182610sf2.pdf]

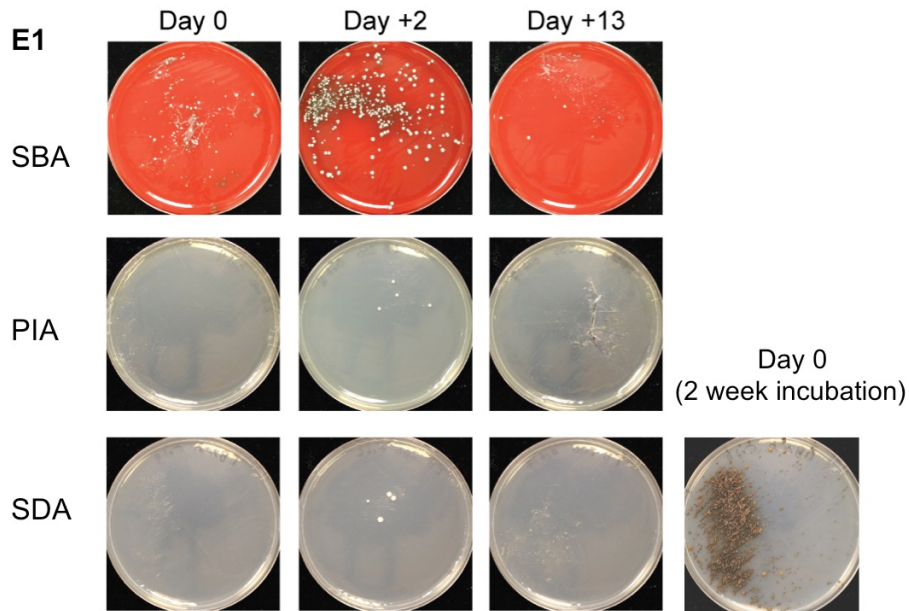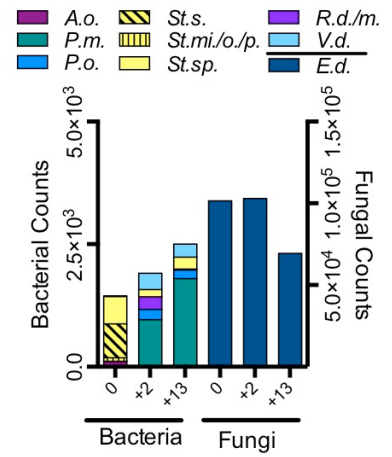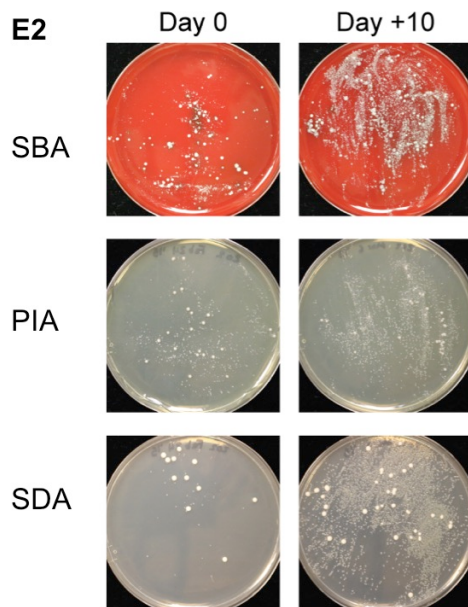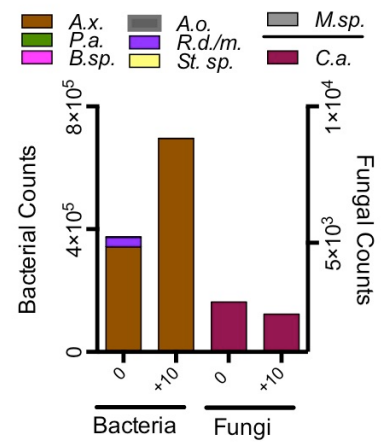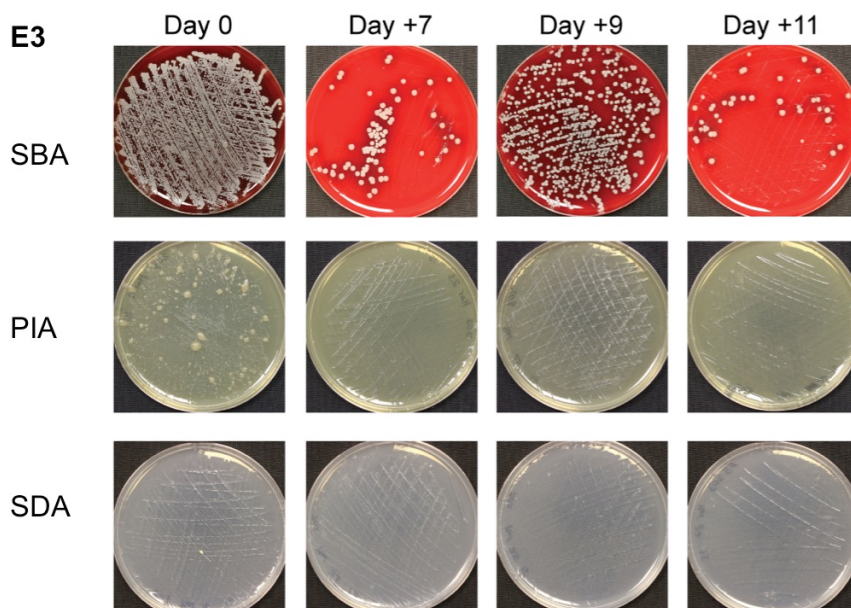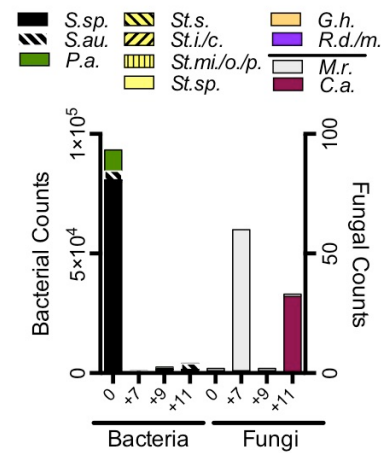

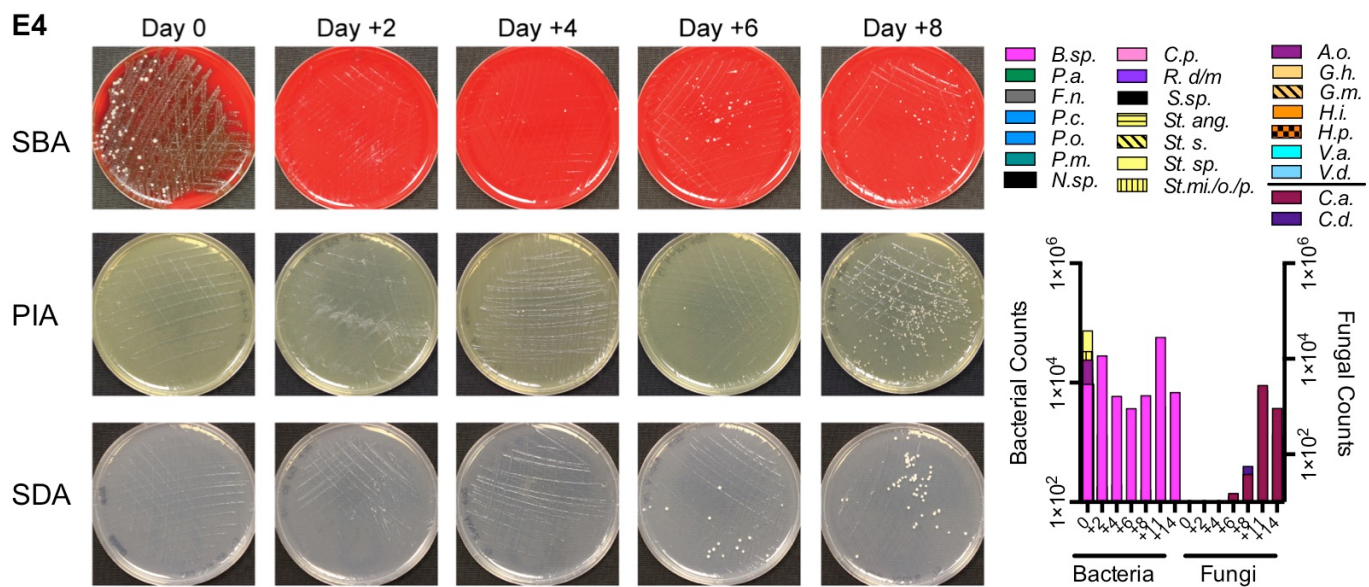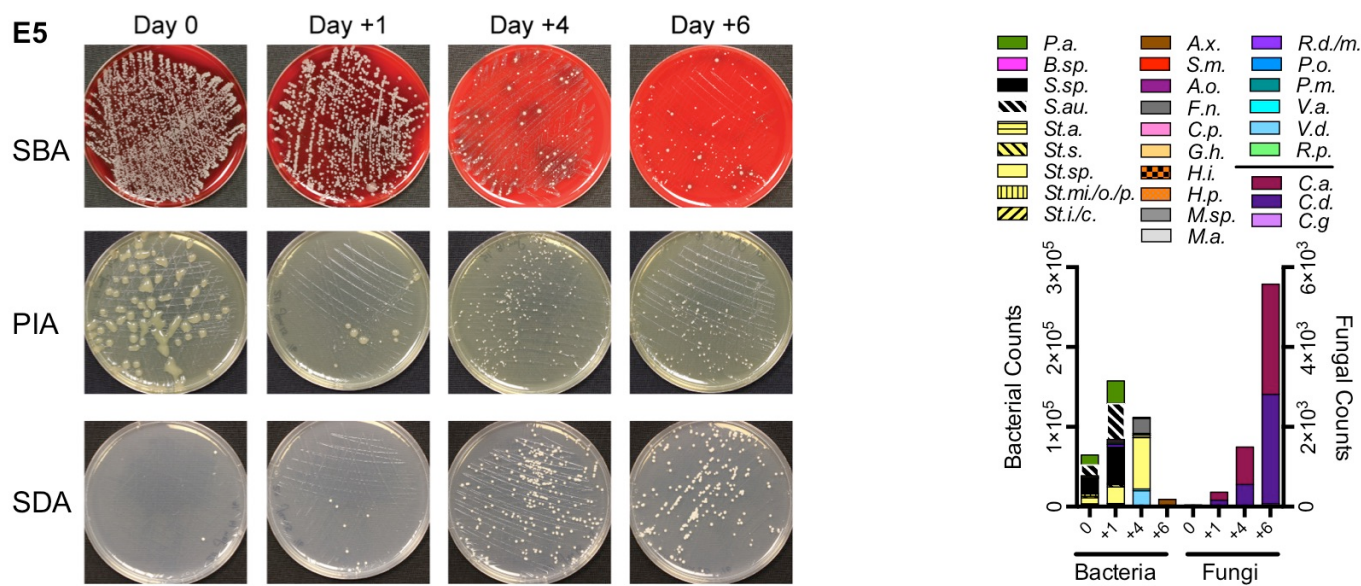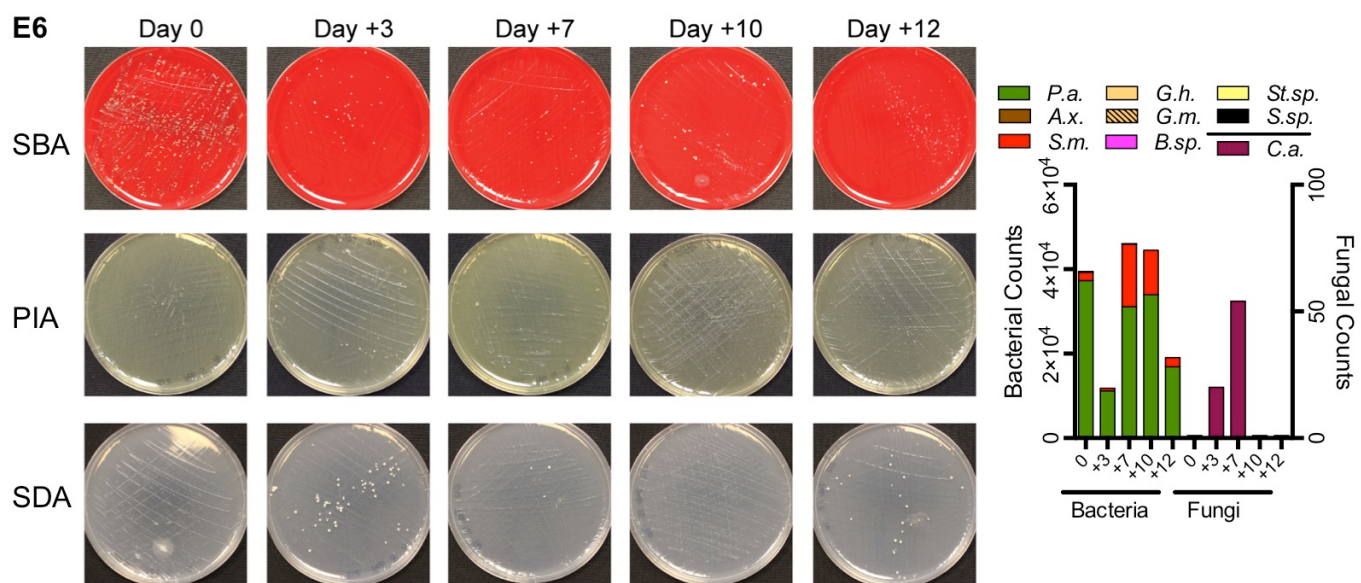

Supplement: FIG S3 [file sph004182610sf3.pdf]
